# Comparison of Nutritional and Functional Components and Antioxidant Activity of Different Foxtail Millet Varieties Grown in the Same Conditions

**DOI:** 10.3390/foods15091516

**Published:** 2026-04-27

**Authors:** Meng Li, Youyang Zhang, Junjie Hao, Runqiang Yang, Lei Luo, Jinle Xiang

**Affiliations:** 1 Faculty of Food & Bioengineering, Henan University of Science & Technology, Luoyang 471023, China; mengli6046@163.com (M.L.); youyang99215@163.com (Y.Z.); 13623896431@139.com (L.L.); 2 Institute of Plant Protection, Henan Academy of Agricultural Sciences, Zhengzhou 450002, China; haojjds@163.com; 3 Faculty of Food Science & Technology, Nanjing Agricultural University, Nanjing 210000, China; yangrq@njau.edu.cn

**Keywords:** foxtail millets, proximate compounds, color characteristics, polyphenols, antioxidant activity

## Abstract

The proximate and phytochemical compounds, antioxidant capacity, and color characteristics of 22 foxtail millet varieties grown in the same location with the same planting conditions and consistent dehulling procedures were compared. The total protein, fat, starch, and resistant starch levels varied significantly (*p* < 0.05) from 8.17 to 11.23 g/100 g, 1.76 to 4.20 g/100 g, 57.69 to 79.64 g/100 g, and 3.15 to 6.53 g/100 g, respectively. The variety JS1 presented the highest total carotenoid content (TCC) of 43.67 mg/kg, together with the highest b* value (b* represents yellowness–blueness). The highest total flavonoid content (TFC) was observed in Yugu 36 (YG36), while Yugu 47 (YG47) displayed the highest total phenolic content (TPC) of 889.57 mg FAE/kg DW. *Trans*-ferulic acid and N′, N″-diferuloylspermidine were detected as the main phenolics in the free phenolic fraction, while the predominate bound phenolic compounds were *trans*-ferulic acid and *trans*-*p*-coumaric acid, ranging from 64.97 to 266.89 mg/kg and 11.76 to 190.74 mg/kg, respectively. The Pearson correlation analysis revealed that b* value was significantly positively correlated with TCC and TPC (*p* < 0.05) while significantly negatively correlated with total starch content (*p* < 0.05). The antioxidant activities were significantly correlated positively with TFC and TPC. The 22 foxtail millets could be classified into three categories by hierarchical clustering analysis (HCA), which exhibited higher TCC and b* value, higher total protein and fat contents, and higher TPC and antioxidant activity, respectively. The heatmap visualization revealed similarities and variations in the color characteristics and phytochemical profiles of the different foxtail millet varieties, and the difference was suggested to be attributed to a gene factor.

## 1. Introduction

Originating in the Yellow River Basin, foxtail millet (*Setaria italica*) has been cultivated as one of the world’s oldest crops [1]. The Food and Agriculture Organization (FAO) declared 2023 as the International Year of Millets to promote it as an alternative crop and address the serious issue of food security and environmental problems in many parts of the world on the United Nations General Assembly (UNGA) [2]. Foxtail millet not only exhibits strong resistance to drought and barrenness but also serves as a vital source of abundant nutrients for humans and diverse phytochemicals with obvious health benefits, including phenolic acids, flavonoids, and carotenoids [3].

The nutritional compositions of foxtail millet have been extensively studied, revealing its high levels of protein, starch, unsaturated fatty acids, minerals, vitamins, and dietary fibers [4]. The nutritional composition of foxtail millet is similar to the three major staple foods (wheat, rice and corn), while foxtail millet contains twice as much total protein and four times as much total fat as rice [5]. Foxtail millet displays a lower amylose level than wheat and corn but a higher percentage of resistant starch [6].

Foxtail millet grain, generally displaying yellow color after being dehusked for consumption, is regarded as an excellent source of natural bioactive compounds. Carotenoids and flavonoids are believed to be the main yellow contributors of dehusked foxtail millets. Among them, carotenoids fight cancer, reduce free radical damage and maintain retinal health [7]. Phenolic acids, mainly including hydroxycinnamic acid and hydroxybenzoic acid derivatives, are important antioxidant compounds in dehusked foxtail millets [8]. These natural phytochemicals assist in reducing chronic and degenerative disorders, such as type 2 diabetes mellitus, cardiovascular diseases, hypertension, and abnormal cholesterol metabolism [9]. In addition, the appearance color, especially yellowness, is reported to closely relate to the accumulation of pigments and antioxidant capacity of some colored cereal grains [10]. However, variations in individual phenolic level and biological activities were widely observed among different foxtail millet varieties, attributed to environmental, cultivation, and genetic factors [11]. When environmental interferences are eliminated, differences in phytochemicals and antioxidant activity of cereals could only be attributed to a genotypic factor.

Metabolomic analysis in combination with heatmap and hierarchical clustering analysis reveals significant differences among multiple foxtail millet varieties from different cultivation regions, which are ascribed to twenty differential metabolites [12]. However, limited information has been reported regarding the correlation between color characteristics and the nutritional and functional compositions and biological activities of various foxtail millets with identical cultivation and processing conditions. The systematic investigation and comparison of phytochemical differences among foxtail millet varieties grown in the same conditions not only help to find the relationship between diverse phytochemical components and certain traits but also enable the screening of superior foxtail millet varieties or with targeted functional requirements. The major objectives of this research were to (1) compare the color characteristics, proximate compound levels, phenolic profiles and antioxidant activities of different dehusked foxtail millet varieties grown in the same cultivation conditions and processed by the same procedures; and (2) explore the relationships among indicators of different foxtail millet varieties by hierarchical clustering analysis and Pearson correlation analysis.

## 2. Materials and Methods

### 2.1. Chemical Reagents and Standards

Folin–Ciocalteu reagent, 6-hydroxy-2,5,7,8-tetramethylchromane-2-carboxylic acid (Trolox), 2′-diphenyl-1-picrylhydrazyl (DPPH), 2,4,6-Tri (2-pyridyl))-s-triazine (TPTZ), and HPLC and MS grade methanol were sourced from Sigma-Aldrich Chemical Co. (St. Louis, MO, USA). All the standards (lutein, protocatechuic aldehyde, *p*-hydroxybenzoic acid, *p*-hydroxybenzaldehyde, syringic acid, *p*-coumaric acid, ferulic acid), 2,2′-azino-bis (3-ethylbenz-othiazoline-6-sulfonic acid) diammonium salt (ABTS)) and *α*-amylase (from pig pancreas) were acquired from Shanghai Yuanye Biotechnology Co., Ltd. (Shanghai, China). Sulphuric acid (H_2_SO_4_), potassium sulphate (K_2_SO_4_), copper sulphate (CuSO_4_), petroleum ether, potassium hydroxide (KOH), sodium hydroxide (NaOH), sodium nitrite (NaNO_2_), sodium carbonate (Na_2_CO_3_), aluminum trichloride (AlCl_3_), ferric trichloride (FeCl_3_), methanol, ethanol, acetone, n-hexane and ethyl acetate were all purchased from Tianjin Dean Chemical Reagent Co., Ltd., (Tianjin, China).

### 2.2. Foxtail Millet Samples and Processing Procedures

A total of 22 foxtail millet varieties planted under consistent field management were harvested from the experimental field of Jinsu Agricultural Technology Co., Ltd., Yichuan county, Henan Province, China, with the GPS coordinates of 112.42, 34.42. The foxtail millet varieties, listed in Table S1, were the main foxtail millet varieties cultivated in northern China. All foxtail millet samples were grown in the cropland with the same ecological conditions in Yichuan County, Luoyang City, and harvested in September 2022. During the growth period of the twenty foxtail millet samples, the experimental field, the soil fertility, and the irrigation were all uniform. The harvested foxtail millet samples were processed by using a separate grain milling machine (6NZF-33, Yutai Quanli Grain Milling Machinery Co., Ltd., Jining, China) through hulling, husk separation, two times milling and polishing processes to remove impurities; dehusked; and polished with the same procedures. The dehusked millet samples were ground, and the sample powder was obtained by a 40-mesh screening and stored in sealed plastic bags at −20 °C.

### 2.3. Determination of Total Protein, Starch, Fat, and Resistant Starch Contents

The contents of total protein, total starch and total fat were determined in accordance with the standard procedures of the American Official Analytical Chemists Association (AOAC) [13]. The total protein content was determined by the Kjeldahl method (AOAC 2001.11) by estimating the total nitrogen content of the defatted samples and multiplying it using a factor of 5.95. The total fat was extracted with petroleum ether based on the Soxhlet method (AOAC 945.16), and the content was quantified via gravimetric measurement. Total starch was degraded into reducing sugars by amylase hydrolysis based on the enzymatic colorimetric method (AOAC 996.11), and the content was calculated based on the detected sugar yield.

The determination of resistant starch (RS) content was conducted using a previously established method with minor adjustments [14]. The sample was firstly digested with pancreatic *α*-amylase and *α*-glucosidase at 37 °C for 16 h after being gelatinized in a boiling water bath for 30 min. The hydrolysate was mixed with anhydrous ethanol. After centrifugation, the precipitate containing resistant starch was obtained by discarding the supernatant. After solubilization of the precipitate using 2 M KOH for 20 min, α-glucosidase was added, and the tubes were placed in a water bath at 50 °C for 30 min. Finally, the glucose content of the supernatants after centrifugation was determined by the 3,5-dinitrosalicylic acid method.

### 2.4. Determination of Total Carotenoid Content

A total of 1 g of foxtail millet powder was mixed with 5 mL of acetone ethanol (1:1, *v*/*v*) and then extracted at 40 °C for 2 h in the dark. The mixture was centrifuged at 10,000 rpm for 10 min, and the supernatant was retained. The total carotenoid contents were measured with lutein as the standard at a wavelength of 450 nm, and the results were calculated and expressed as mg/kg [10].

### 2.5. Measurement of Color Parameters

The color variations of the dehusked foxtail millet varieties differed with L* (lightness), a* (from green to red), b* (from blue to yellow), and c* (saturation value) by the colorimeter (Color i5, Xrite, Grand Rapids, MI, USA).

### 2.6. Determination of Total Phenolic Content and Total Flavonoid Content

Extractions of soluble free and insoluble bound phenolic compounds were conducted according to our described procedure [8]. The dehusked foxtail millet powder was defatted twice with hexane and then extracted with 80% methanol for 60 min. After centrifugation, the supernatant was collected, and the sample was extracted once again. After methanol extraction, the remaining residue was hydrolyzed with a 2 M NaOH solution for 2 h at room temperature, followed by adjustment of pH to 1.5 to 2.0 with 6 M HCl. After centrifugation, the hydrolysate was extracted with ethyl acetate three times to obtain the bound phenolic extract. The liquid fraction was dried by rotary evaporation and dissolved in 50% methanol solution as free and bound phenolic extracts.

The total phenolic content (TPC) was determined by using the Folin–Ciocalteau colorimetric method. A 96-well microplate reader (PT06-96S, Bioland Biotechnology Co., Ltd. Hangzhou, China) was used to determine the TPC of the extracts using our reported method [15]. The sample extract (20 μL) was mixed with Folin–Ciocalteu reagent (40 μL), followed by the addition of 160 μL of 75 g/L Na_2_CO_3_. After reacting for 1.5 h in the dark, the absorbance was measured at 750 nm. Ferulic acid was used as the standard, and TPC was expressed as milligrams of ferulic acid equivalents per kilogram of dry weight (mg FAE/kg DW).

The total flavonoid content (TFC) of the free phenolic extract was measured by our previously described AlCl_3_ colorimetric method [16]. The appropriately diluted sample extract was mixed with 75 μL of NaNO_2_ (5%), 150 μL of AlCl_3_ (10%) was added after 5 min, and 0.5 mL of NaOH (4%) and pure water were then added to obtain a final volume of 2.5 mL. After reaction in the dark for 15 min, absorbance was read at 510 nm. Rutin was applied as the standard, and the TFC was expressed as mg of Rutin equivalents per kg (mg RE/kg).

### 2.7. Analysis of Individual Polyphenols

The UPLC coupled to a QQQ-MS (Waters Xevo TQ-S/micro) was employed for analysis. A C_18_ column (100 mm × 3 mm, Thermo Fisher Scientific, Waltham, MA, USA) was used. The binary-mobile phase system was composed of solvent A (water containing 0.1% formic acid) and solvent B (methanol containing 0.1% formic acid). A flow rate of 0.4 mL/min was used and was run in a 25 min gradient elution program. The elution procedure and QQQ-MS conditions were set the same as our previously described parameters [9]. All the individual polyphenols were identified by UPLC-MS^2^.

An external standard method was used to calculate the individual phenolic levels. Protocatechuic aldehyde, *p*-hydroxybenzaldehyde, *p*-hydroxybenzoic acid, and syringic acid were determined at 280 nm. Ferulic acid and *p*-coumaric acid were detected at 320 nm. The contents of individual phenolics were calculated and expressed as mg per kg dry weight (mg/kg DW).

### 2.8. Determination of Antioxidant Activity In Vitro

DPPH radical scavenging activity was measured as described in [8]. The phenolic extract (10 μL) was added and thoroughly mixed with DPPH radical working solution (190 μL). After incubating under dark conditions at room temperature for 30 min in a 96-well microplate, the absorbance was measured at 515 nm using a microplate reader (BIO-RAD Instruments Inc., Hercules, CA, USA). Trolox was used as the standard, and the results were calculated as micromole Trolox equivalents per gram of dry weight (μmol TE/g DW).

The ABTS^+^ assay was based on our reported method using a 96-well microplate reader [8]. The ABTS^·+^ stock solution was diluted with ethanol to obtain a working solution with an initial absorbance of approximately 0.80 at 750 nm. The phenolic sample (10 μL) was added and mixed thoroughly with ABTS^+^ radical working solution (190 μL). After incubation for 30 min under dark conditions, the absorbance was measured at 750 nm. A standard curve of Trolox was obtained, and the results were calculated as μmol TE/g DW.

Determination of FRAP of the phenolic extract was conducted with the previously described method [17]. The phenolic extract (10 μL) was reacted with 300 μL of ferric TPTZ reagent, which was a mixture of 300 mM acetate buffer, 100 mM TPTZ in a 40 mM hydrochloric acid solution, and a 20 mM FeCl_3_·6H_2_O solution in a volume ratio of 10:1:1. The absorbance of the reaction system at 593 nm was measured after incubation for 30 min. A standard curve using Trolox was drawn, and the FRAP value was expressed as μmol TE/g DW.

### 2.9. Statistical Analysis

All the analyses were carried out at least in triplicate, and the results were reported as mean ± standard deviation (SD). Differences of mean values among different phenolic fractions of foxtail millet were analyzed using ANOVA, followed by Tukey’s HSD test, at a *p* < 0.05 significance level. Violin plots, Pearson correlation analyses, and HCA were performed by Origin 2021 (Origin Lab, Inc., Northampton, MA, USA). All the data were processed by IBM SPSS Statistics (Version 26, IBM Corp., Armonk, NY, USA).

## 3. Results and Discussion

### 3.1. The Proximate Compound Levels of the Dehusked Foxtail Millet Varieties

**Figure 1:**
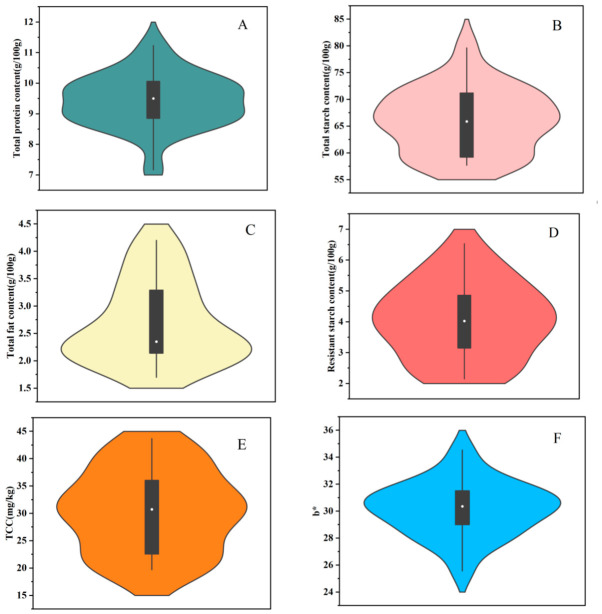
Violin plots of proximate compound levels and color parameters of 22 varieties of dehusked foxtail millet. (**A**) Total protein content; (**B**) total starch content; (**C**) total fat content; (**D**) total resistant starch content; (**E**) total carotenoid content (TCC); (**F**) b* value.

The total protein and total starch contents of the dehusked foxtail millet varieties are displayed in violin plots in Figure 1A,B, which show the widest outline ranging from 8.54 to 10.02 and 58.96 to 72.80 g/100 g, respectively, indicating that most of the varieties concentrated the total protein and starch contents within these ranges. Similarly, the nutritional compositions of different varieties of the dehulled foxtail millet from Hebei Province of China were measured, with starch and protein content ranging from 72.9% to 81.4% and 8.9% to 16.0%, respectively [18].

The total fat contents of the 22 dehusked foxtail millets are presented in Figure 1C. Most dehusked foxtail millets predominantly concentrated the total fat level from 2.09 to 3.80 g/100 g. This result is substantially consistent with the previously reported data of foxtail millet varieties from different ecological regions of Shanxi Province of China, ranging from 3.1% to 3.8% [3]. The total protein, total starch, and total fat contents of the 22 dehusked foxtail millets are provided in Table S2, and significant differences (*p* < 0.05) were observed. Among them, variety JG39 showed the highest protein level, and variety JG48 displayed the highest fat content, while the highest total starch content was observed in variety YG18. These differences are likely to be mainly caused by the genetic variations of the foxtail millet varieties due to the consistency of planting conditions and processing procedures.

The resistant starch level of the foxtail millet varieties is displayed in Figure 1D, which shows that most millets concentrated the content from 3.15 to 4.86 g/100 g, while the foxtail millet varieties of HG11, HG36, CG9, and BG928 showed obviously higher resistant starch content, with a mean value of 5.51 g/100 g. It could be compared that the dehusked foxtail millets contain higher resistant starch than other cereal grains, such as sweet corn, with resistant starch of 1.16 g/100 g, and milled rice, with resistant starch 2.72 g/100 g [19]. Our results suggest that dehusked foxtail millets may serve as a great potential food source of resistant starch.

### 3.2. Color Characteristics of the Dehusked Foxtail Millets

The color parameters of the dehusked millets were determined and are reflected by L*, a*, b*, and c* values, and the data are provided in Table S1. Significant differences (*p* < 0.05) were observed with respect to the L*, a*, b*, and c* values of the dehusked foxtail millets. Variety ZZ36 exhibited the maximum L* value, with the highest lightness, while HG36 showed the lowest lightness. The a* value indicated variation from red (positive) to green (negative), and the a* value of the 22 foxtail millet varieties narrowly ranged from 3.57 to 5.48.

In general, yellow color is the simplest indicator for consumers to appraise the quality of commercial foxtail millets. The b* value of the dehusked foxtail millets displayed a wide range between 29.00 and 31.77, and the results are shown in Figure 1F. Variety ZZ36 showed the lowest b* value of 25.57, while JS1 displayed the highest value, up to 34.53. Similarly, Li et al. [20] reported a wider range of b* values from 17.74 to 40.65 for foxtail millets cultivated in Hebei Province of China.

### 3.3. Variation in Carotenoid Level

The total carotenoid content (TCC) of the 22 dehusked foxtail millet samples is presented in Table S2. The TCC ranged from the lowest value of 19.71 mg/kg DW to the highest value of 43.67 mg/kg DW, with the mean level of 30.57 mg/kg DW. As seen in the violin plot of TCC shown in Figure 1E, the widest outline was observed between 28.19 and 33.71 mg/kg DW, demonstrating that most of the millet varieties concentrated their TCC levels within this range. The variety JS1 presented the highest TCC and concurrently displayed the highest b* value. It could be hypothesized that the b* value of dehusked foxtail millet was correlated positively with TCC. The overwhelming majority of the dehusked foxtail millets showed higher TCC than those of previously reported foxtail millet varieties, ranging from 12.98 to 22.55 mg/kg [7]. Our results also showed that the TCC of the dehusked foxtail millets were comparable with those of yellow maize, ranging from 15.94 to 66.46 mg/kg, which is believed to be one of the most abundant carotenoid-rich cereal food sources [21]. The dehusked foxtail millets could also be another important cereal food source of dietary carotenoids for human health.

### 3.4. Variations in TFC and TPC

The TFC and TPC of the 22 dehusked foxtail millets are shown in Table 1. The TFC of foxtail millet varieties ranged widely from 90.52 to 237.75 mg RE/kg DW, with the mean value of 121.17 mg RE/kg DW. Among them, twelve varieties exhibited no significant difference (*p* > 0.05) in TFC. Our results were substantially consistent with those of previously determined foxtail millet cultivars grown in China [22]. YG36 presented the highest TFC, which was 2.63 times higher than JG42, which had the lowest value. However, this lowest value of total flavonoids was still significantly higher than those of barley and yellow maize [10,23], indicating that the dehusked foxtail millets may be a valuable cereal source of flavonoids.

The free TPC of dehusked foxtail millet ranged from 149.35 to 333.70 mg FAE/kg DW, which was correspondingly lower than the bound TPC spanning from 264.13 to 584.13 mg FAE/kg DW. Bound TPC accounted for 55.75–75.78% of the total TPC (sum of soluble free and insoluble bound phenolic extract). Similar performance had been found in dehusked proso millets that bound TPC, contributing 62.08–67.05% of total TPC [24]. Phenolic acids in most cereal grains mainly exist in bound form, linked to cellulose, lignin and proteins through ester bonds [25].

The total TPC of the 22 foxtail millet varieties ranged from 413.48 mg FAE/kg DW to 889.57 mg FAE/kg DW, which was higher than the reported TPC of four dehusked foxtail millets from India ranging from 272.20 to 505.30 mg GAE/kg DW [26]. In addition to the difference in genotype, the phenolic levels of millets are generally affected by many factors, such as growing environment, water and fertilizer management, and processing technology [27]. Since the growth environment, cultivation conditions, and processing conditions for the dehusked foxtail millets were the same in this study, the variations in the phenolic level of the dehusked foxtail millets could only be attributed to a genetic factor.

### 3.5. Phenolic Profiles and Main Individual Phenolic Compound Contents

**Figure 2:**
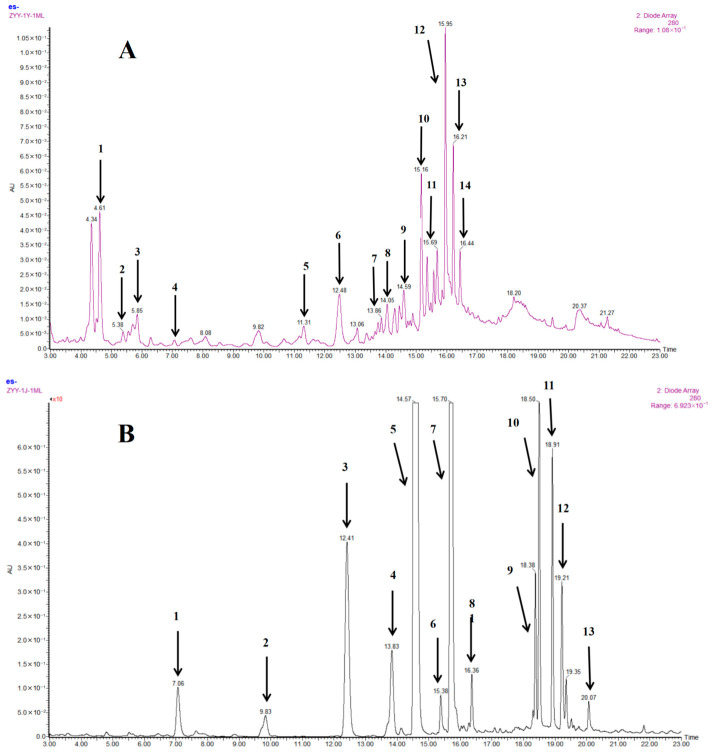
UPLC chromatograms of soluble free (**A**) and insoluble bound (**B**) phenolics from JS1 foxtail millet. Detection is set at a wavelength of 280 nm. Peaks were confirmed by comparison with authentic standards when available or attentively identified by UV spectra, MS and MS/MS data. Phenolic compounds correspond to peak numbers, (**A**) 1: protocatechuic aldehyde. 2: *p*-hydroxybenzoic acid. 3: syringic acid. 4: *p*-hydroxybenzaldehyde. 5: 4-*p*-coumaroylquinic acid. 6: *trans*-*p*-coumaric acid. 7: feruloylquinic acid. 8: 1-*O*-*p*-coumaroylglycerol. 9: *trans*-ferulic acid. 10: N′-caffeoylspermidine. 11: N′, N″-di-p-coumaroylspermidine. 12: Kaempferol-*C*, *O*-dihexoside. 13: N′-*p*-coumaroyl-*N*″-feruloylspermidine. 14: N′, N″-diferuloylspermine.; (**B**) 1: *p*-hydroxybenzaldehyde. 2: syringic acid. 3: *trans*-*p*-coumaric acid. 4: *cis*-*p*-coumaric acid. 5: *trans*-ferulic acid. 6: 8,8′-aryltetralin-DFA. 7: *cis*-ferulic acid. 8: 8,5′-DFA. 9: *trans*-*trans*-8-*O*-4′-DFA. 10: TFA. 11: 8,8′-DFA. 12: 8-5′-DFA benzofuran. 13: *trans*-*cis*-8-*O*-4′-DFA. DFA: ferulic acid dimer. TFA: ferulic acid trimer.

The fingerprint chromatograms (shown in Figure S1) of the free and bound phenolics from the 22 millet varieties were respectively consistent in shape, which indicated that the different millet varieties showed no obvious difference in phenolic composition. Figure 2 depicts the soluble free (A) and insoluble bound (B) phenolic profiles of the presentative chromatograms of the dehusked millet variety. Fourteen free phenolic compounds and thirteen bound phenolic acids were identified by analyzing their MS^2^ fragment information and UV spectral characteristics and then comparing with standards or our previously reported results [28].

#### 3.5.1. Soluble Free Phenolic Compounds

The contents of the main individual free phenolic compounds in the 22 dehusked foxtail millets are shown in Table 2. Significant differences (*p* < 0.05) were observed in all individual free phenolic compounds among the 22 varieties. For the foxtail millet varieties, the free phenolics were predominately composed of hydroxycinnamic acids and their spermidine conjugated derivates, including *trans*-ferulic acid, ranging from 1.33 to 5.69 mg/kg, and *trans*-*p-*coumaric acid, ranging from 0.41 to 1.00 mg/kg. Among them, *trans*-ferulic acid was the most abundant phenolic acid, and variety TS6 showed the highest level. Chandrasekara and Shahidi [25] reported that *p*-coumaric acid, ferulic acid and syringic acid were the major free phenolics existing in different foxtail millet varieties. In this study, syringic acid was detected not in major, ranging from 0.13 mg/kg to 0.41 mg/kg. Similarly, *trans-p*-coumaric acid also varied significantly among varieties, with JS1 exhibiting a relatively high level, revealing its characteristic phenolic composition.

The three hydroxycinnamic acid amides (HCAAs), namely N′, N″-*di*-*p*-coumaroylspermidine, N′-*p*-coumaroyl-*N*″-feruloylspermidine and N′, N″-diferuloylspermine, were observed presenting in all 22 dehusked foxtail millet varieties, and N′, N″-diferuloylspermine was detected as the main HCCA component, ranging from 0.92 to 5.56 mg/kg. JG42 had the highest content of N′, N″-diferuloylspermidine and could be regarded as a typical variety rich in HCAAs. The level of N′, N″-di-*p*-coumaroylspermidine ranges from 0.36 to 2.27 mg/kg, with ZG989 displaying the highest level, and N′-*p*-coumaroyl-*N*″-feruloylspermidine ranges from 0.35 to 1.22 mg/kg, with JG20 displaying the highest value. Given that all the millet samples analyzed in this study were cultivated under identical ecological conditions, the observed HCAA variations could be attributed solely to their distinct genotypes.

#### 3.5.2. Insoluble Bound Phenolic Compounds

The levels of the individual bound phenolics in the dehusked foxtail millets are shown in Table 3. *Trans*-ferulic acid was detected as the most abundant phenolic component in bound form, ranging from 64.97 to 357.54 mg/kg DW. The variety YG47 contained the highest level of *trans*-ferulic acid, meanwhile presented the highest bound TPC of 555.87 mg FAE/100 g DW. On the contrary, the variety JS1 displayed the lowest *trans*-ferulic acid level, corresponding to the lowest bound TPC of 264.13 mg FAE/100 g DW. These performances indicated that *trans*-ferulic acid contributed substantially to the bound TPC of the dehusked foxtail millets. The results were consistent with the previous report on bound TPC of different dehusked foxtail millets collected from four main regions of China [8].

As a common phenolic acid in cereal grains, *trans*-*p*-coumaric acid is commonly existing in both soluble free and insoluble bound forms in foxtail millets. The content of insoluble bound *trans*-*p*-coumaric acid in the foxtail millets ranged from 11.76 to 190.74 mg/kg DW, which was far higher than that in soluble free form. For example, the level of bound *trans*-*p*-coumaric acid in variety YG18 was about 225-fold as that in soluble free form. In agreement with our results, previous research also reported the contents of bound ferulic acid and *p*-coumaric acid were far higher than those in soluble free form in dehulled foxtail millet and little millet from India [29]. Except for varieties JG42 and JG39, bound *cis*-*p*-coumaric acid was detected in the other 20 foxtail millet varieties in minor, ranging from 2.52 to 6.85 mg/kg DW. The *p*-hydroxybenzaldehyde in bound was existing in minor, ranging from 0.39 to 082 mg/kg DW, while syringic acid was only present in the 14 selected foxtail millet varieties, ranging from 0.29 to 0.92 mg/kg DW.

Ferulic acid dehydrodimers (DFAs) are phenolic acid derivatives formed by the oxidative coupling of two ferulic acid molecules and are important phytochemicals for maintaining the stability of plant cell walls [30]. In this study, six individual DFAs were detected in minor amounts because DFAs predominantly present in the husk and bran layers of foxtail millet grains [31], which had been mostly removed by the dehull process. Therefore, their total amount was calculated, ranging from 0.72 to 20.36 mg/kg DW.

### 3.6. Antioxidant Properties

The antioxidant capacities of the free and bound phenolic extracts were comprehensively evaluated by three independent assays, including DPPH, ABTS and FRAP. The results are summarized in Table 4.

The DPPH radical scavenging activity of the dehusked foxtail millet varieties of free and bound extracts ranged from 0.46 to 0.92 μmol TE/g DW and 0.83 to 1.51 μmol TE/g DW, respectively. The variety CG9 showed the highest DPPH values for both free and bound phenolics, which can probably be attributed to the correspondingly higher free and bound phenolic values of 235.43 mg FAE/kg DW and 561.96 mg FAE/kg DW.

The ABTS radical scavenging activity of free and bound extracts ranged from 0.51 to 1.79 μmol TE/g DW and 0.82 to 2.37 μmol TE/g DW, respectively. The variety YG47 displayed the highest ABTS value for the free phenolics, and JG48 showed the highest ABTS value for the bound phenolics. The total ABTS values of varieties YG47 and JG48 were 3.88 and 3.78 µmol TE/g DW respectively, and their performance was superior to that of other varieties.

For the FRAP assay, HG36 and HG12 exhibited the highest values of 1.19 μmol TE/g DW and 1.16 μmol TE/g DW, respectively, for their free form phenolics, while JG48 and YG36 were preferable in bound phenolics, with the higher FRAP values of 2.06 μmol TE/g DW and 1.79 μmol TE/g DW, respectively. Total FRAP activity peaked in HG36 and YG47, with the values of 2.85 μmol TE/g DW and 2.97 μmol TE/g DW, respectively, reflecting the highest reducing antioxidant capacity. Consistent with TPC of the foxtail millets, the bound phenolics provided higher contributions than their corresponding free phenolics. For the total antioxidant capacity of the foxtail millet varieties, no single variety excelled across all three assays, probably due to the different antioxidant mechanisms. The variety YG47 exhibited the highest total ABTS and FRAP values, while CG9 exhibited the highest total DPPH value. Our results showed that the antioxidant capacity of dehusked foxtail millet was obviously higher than that of refined wheat flour [32]. Especially, some foxtail millet varieties, such as CG9 and YG47, exhibited high levels of antioxidant activity, suggesting that they may be ideal sources of phenolic antioxidants.

### 3.7. Correlation Analysis

**Figure 3:**
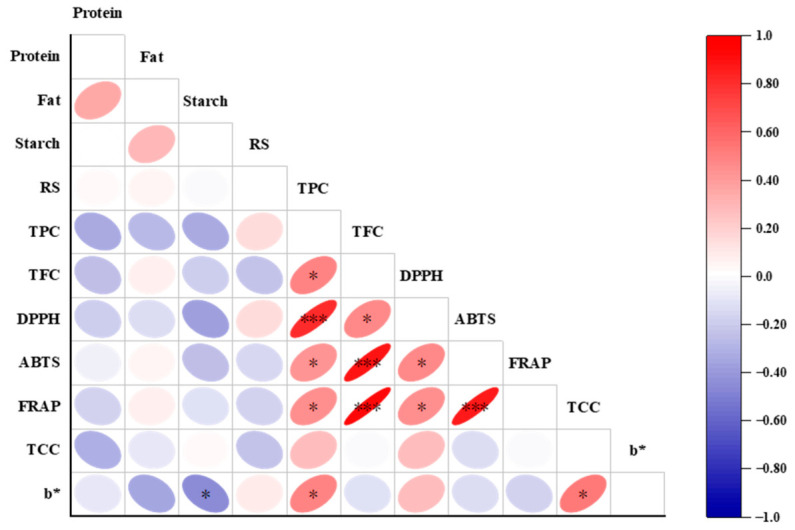
Pearson correlation heatmap of nutritional compositions, phytochemicals and antioxidant activities of 22 foxtail millet varieties. Red and blue mean positive and negative correlation, respectively. Meanwhile, the darker the color and the flatter the ellipse, the stronger the correlation, while the correlation is not significant when the color is light and the ellipse is close to a circle. The correlation significance level is set as * represents *p* < 0.05, and *** represents *p* < 0.001. RS: resistant starch.

The relationships among the levels of proximate compounds, TCC, TFC, TPC, color parameters, and total antioxidant capacity of foxtail millets were investigated using Pearson correlation analysis, with the results shown in Figure 3. The DPPH scavenging activity was significantly (*p* < 0.05) correlated positively with TFC and displayed an extremely significant (*p* < 0.001) positive correlation with TPC. The ABTS and FRAP values showed significant (*p* < 0.05) positive correlation with TPC and exhibited extremely significant (*p* < 0.001) positive correlations with TFC, respectively. The aforementioned results show that foxtail millet varieties with higher antioxidant capacity presented relatively elevated TPC or TFC. Similarly, significant (*p* < 0.01) correlations between TPC and antioxidant capacity measured by the DPPH, ABTS and FRAP assays were reported in phenolic extracts from finger millets [33].

Color characteristics not only play an important role in simple and direct assessment of food quality but also are one of the potential indicators that can be used to evaluate food ingredients. Previous research observed that the b* value of durum wheat was significantly positively correlated with TCC [34]. It was also reported that the b* value reflecting yellow degree of yellow maize varieties was significantly correlated with TCC (*p* < 0.01); meanwhile, the b* value displayed extremely significant (*p* < 0.001) correlations with TPC, TFC, and antioxidant properties in the DPPH, ABTS and FRAP assays [10]. In this research, the b* value of the dehusked foxtail millets exhibited significantly positive correlation with TCC and TPC, respectively (*p* < 0.05).

However, significantly negative correlation (*p* < 0.05) was observed between total starch content and b* value, which was probably because the dehulling and polishing process resulted in the loss of some yellow color contributors (such as carotenoids, vitamin B2, and flavonoids) existing in the aleurone layer, while the starch in the endosperm of foxtail millet grain was entirely retained [4]. The whole weight of foxtail millet grains decreased by the dehulling and polishing process, while the weight of starch remained unchanged, which resulted in an increase in the total starch percentage. Our findings suggest that the b* value of color parameters could be used as a simple indicator for some nutritional and functional components of dehusked foxtail millets.

### 3.8. Cluster Analysis

**Figure 4:**
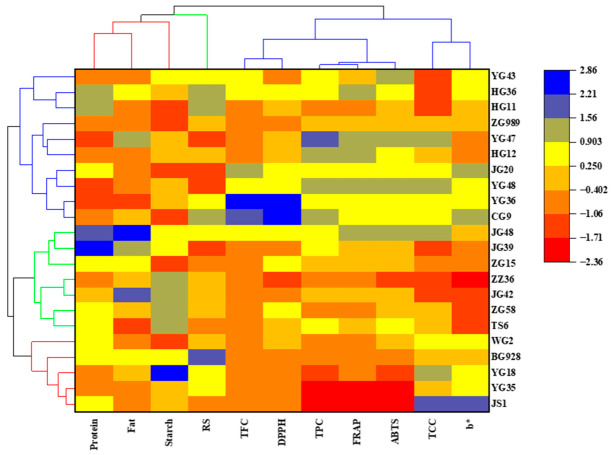
Heatmap and hierarchical cluster analysis of 22 foxtail millet varieties based on nutritional compositions, phytochemicals, and antioxidant activities. The change in color of the scale from red to blue indicates the level from low value to high value.

Hierarchical clustering analysis (HCA) is widely used for the comprehensive evaluation of different variety samples, and samples with high similarity are classified into one class, which can provide a certain reference for selecting excellent varieties. The 22 varieties of foxtail millet were categorized into three categories by HCA, and the results are shown in Figure 4.

The varieties of YG18, YG35, JS1 BG928, and WG2 were classified into the first category, which presented higher TCC and b* value. Seven foxtail millet varieties were classified into the second category, which exhibited higher contents of total protein and crude fat, with a lower b* value. The last ten varieties were classified into the third category, which showed higher TPC and TFC and higher values of ABTS, FRAP and DPPH.

It was worth noting that foxtail millet varieties abbreviated as the same upper case with different numbers, representing the same origin, were grouped together by the hierarchical cluster analysis. It was probably because these foxtail millet varieties were bred from the same pistillate parent millet with consistent original genotype [35]. HCA facilitated comprehension of the similarities and differences in color parameters and nutrient compositions of different foxtail millet varieties grown in the same geographical location and ecological environment and provided reference for breeding varieties with specified nutritional and functional requirements.

## 4. Conclusions

The proximate compound levels, including total protein, crude fat, total starch, and resistant starch, varied significantly (*p* < 0.05) among the 22 dehusked foxtail millet varieties. Significant differences were also observed in the phytochemical levels, including total carotenoid, phenolic and flavonoid contents, together with their antioxidant properties assayed by the DPPH, ABTS and FRAP systems. The different foxtail millets displayed similar free and bound phenolic profiles, with *trans*-ferulic acid and N′, N″-diferuloylspermidine being the major phenolic compounds in free form, and *trans*- and *cis*-ferulic acid, *trans-p*-coumaric acid being the predominant phenolics in bound. Pearson correlation analysis revealed that the b* value showed positive correlations with TCC and TPC and a negative correlation with total starch content (*p* < 0.05). The antioxidant activities were significantly correlated positively with TFC and TPC. The 22 foxtail millet varieties were classified into three categories through hierarchical clustering analysis. The first category presented higher TCC and b* value, the second category exhibited higher total protein and fat contents with lower b* value, and the third category showed higher TPC, TFC, and antioxidant properties. The findings could provide consumers and breeding experts some references for screening foxtail millet varieties with more targeted nutritious and functional compounds.

## Figures and Tables

**Table 1:** Total phenolic contents (TPC) and total flavonoid contents (TFC) of the 22 dehusked foxtail millet varieties.

Dehusked Foxtail Millets	TFC (mg RE/kg DW)	TPC (mg FAE/kg DW)		
		Free	Bound	Total
JS1	90.83 ± 5.01 ^fg^	149.35 ± 25.39 ^l^	264.13 ± 1.99 ^jk^	413.48 ± 27.38 ^m^
WG2	103.63 ± 4.26 ^f^	215.43 ± 8.49 ^hij^	326.74 ± 16.96 ^i^	542.17 ± 25.45 ^jk^
TS6	88.57 ± 6.27 ^g^	286.74 ± 14.31 ^bc^	405.00 ± 26.05 ^gh^	691.74 ± 40.36 ^efg^
CG9	202.68 ± 8.53 ^b^	235.43 ± 14.11 ^efgh^	561.96 ± 3.98 ^ab^	797.39 ± 18.09 ^bc^
HG11	99.77 ± 3.28 ^fg^	158.48 ± 27.24 ^kl^	433.26 ± 22.45 ^fgh^	591.74 ± 49.69 ^hij^
HG36	150.58 ± 5.69 ^de^	188.48 ± 5.69 ^ijk^	515.43 ± 17.22 ^bcd^	703.91 ± 22.91 ^efg^
HG12	101.22 ± 13.27 ^fg^	270.22 ± 17.99 ^bcd^	584.13 ± 34.05 ^a^	854.35 ± 52.04 ^ab^
ZG15	95.28 ± 3.84 ^fg^	227.17 ± 18.28 ^fgh^	383.70 ± 2.72 ^h^	610.87 ± 21.00 ^hij^
ZG989	96.65 ± 3.27 ^fg^	221.52 ± 18.32 ^ghi^	420.22 ± 1.99 ^gh^	641.74 ± 20.31 ^ghi^
YG18	100.22 ± 6.08 ^fg^	258.04 ± 13.95 ^cdef^	245.03 ± 3.98 ^k^	503.07 ± 17.93 ^kl^
YG35	101.52 ± 5.58 ^fg^	184.57 ± 18.92 ^jk^	243.70 ± 10.68 ^k^	428.27 ± 29.60 ^lm^
YG36	237.75 ± 6.89 ^a^	292.83 ± 15.81 ^b^	451.96 ± 21.94 ^efg^	744.79 ± 37.75 ^cde^
YG43	137.93 ± 6.29 ^de^	264.57 ± 6.43 ^bcde^	455.43 ± 24.55 ^efg^	720.00 ± 30.98 ^def^
YG47	102.78 ± 5.05 ^f^	333.70 ± 21.45 ^a^	555.87 ± 3.28 ^ab^	889.57 ± 24.73 ^a^
YG48	157.14 ± 5.41 ^d^	252.83 ± 17.61 ^defg^	529.35 ± 21.09 ^bc^	782.18 ± 38.70 ^bcd^
JG20	177.93 ± 6.03 ^c^	228.93 ± 11.59 ^fgh^	531.09 ± 41.17 ^bc^	760.02 ± 52.76 ^cde^
JG39	91.18 ± 4.69 ^fg^	223.26 ± 5.43 ^gh^	471.52 ± 55.77 ^def^	694.78 ± 61.20 ^efg^
JG42	90.52 ± 4.26 ^fg^	158.91 ± 7.18 ^kl^	497.17 ± 36.90 ^cde^	656.08 ± 44.08 ^fgh^
JG48	141.03 ± 3.28 ^e^	211.96 ± 13.24 ^hij^	524.13 ± 15.29 ^bc^	736.09 ± 28.53 ^cde^
ZG58	99.67 ± 4.69 ^fg^	183.68 ± 19.97 ^jk^	386.30 ± 44.55 ^h^	569.98 ± 64.52 ^ijk^
ZZ36	91.19 ± 5.69 ^fg^	145.87 ± 15.72 ^l^	391.52 ± 16.97 ^h^	537.39 ± 32.69 ^jk^
BG928	97.44 ± 4.69 ^fg^	241.09 ± 10.54 ^defgh^	303.70 ± 6.69 ^ij^	544.79 ± 17.23 ^jk^

Values with no letters in common are significantly different (*p* < 0.05). DW, dry weight of sample. Results are expressed as mean ± SD.

**Table 2:** Contents of individual phenolic compounds (mg/kg DW) in free phenolic extracts of 22 dehusked foxtail millet varieties.

Dehusked Foxtail Millets	Protocatechuic Aldehyde	*p*-Hydroxybenzoic Acid	Syringic Acid	*Trans*-*p*- Coumaric Acid	1-O-*p*- Coumaroylg-Lycerol	*Trans*- Ferulic Acid	N′, N″-di-*p*- Coumaroylsp- Ermidine	N′-*p*-Coumaroyl-N″-Ferulo-Ylspermidine	N′, N″- Diferuloylspe-Rmine
JS1	0.21 ± 0.01 ^g^	0.12 ± 0.01 ^fgh^	0.41 ± 0.01 ^a^	1.00 ± 0.08 ^a^	0.75 ± 0.04 ^a^	2.46 ± 0.33 ^ghi^	1.55 ± 0.05 ^def^	0.94 ± 0.02 ^e^	2.13 ± 0.05 ^ef^
WG2	0.11 ± 0.01 ^l^	0.13 ± 0.01 ^fg^	0.20 ± 0.02 ^gh^	0.65 ± 0.02 ^fg^	0.30 ± 0.01 ^hi^	2.30 ± 0.08 ^ij^	1.08 ± 0.06 ^h^	0.44 ± 0.06 ^k^	1.19 ± 0.14 ^mn^
TS6	0.26 ± 0.03 ^hi^	0.19 ± 0.02 ^bc^	0.20 ± 0.01 ^fg^	0.89 ± 0.04 ^bc^	0.28 ± 0.01 ^ij^	5.69 ± 0.23 ^a^	1.66 ± 0.05 ^cde^	0.36 ± 0.01 ^l^	3.15 ± 0.08 ^c^
CG9	0.25 ± 0.02 ^i^	0.10 ± 0.01 ^hi^	0.35 ± 0.03 ^b^	0.72 ± 0.01 ^ef^	0.27 ± 0.01 ^j^	1.94 ± 0.07 ^k^	0.36 ± 0.02 ^j^	0.49 ± 0.02 ^jk^	1.83 ± 0.06 ^gh^
HG11	0.42 ± 0.01 ^d^	0.15 ± 0.02 ^ef^	0.25 ± 0.01 ^de^	0.68 ± 0.01 ^fg^	0.36 ± 0.01 ^e^	2.85 ± 0.14 ^ef^	1.43 ± 0.06 ^fg^	0.85 ± 0.01 ^fg^	1.20 ± 0.04 ^g^
HG36	0.11 ± 0.01 ^l^	0.10 ± 0.01 ^hi^	0.22 ± 0.01 ^fg^	0.54 ± 0.02 ^h^	0.35 ± 0.01 ^efg^	2.28 ± 0.04 ^ij^	0.86 ± 0.13 ^i^	0.58 ± 0.01 ^i^	1.32 ± 0.04 ^klm^
HG12	0.32 ± 0.01 ^g^	0.20 ± 0.03 ^b^	0.30 ± 0.01 ^c^	0.65 ± 0.04 ^fg^	0.31 ± 0.01 ^h^	4.42 ± 0.25 ^b^	0.42 ± 0.01 ^j^	0.48 ± 0.02 ^k^	0.92 ± 0.06 ^o^
ZG15	0.15 ± 0.01 ^k^	0.23 ± 0.02 ^b^	0.23 ± 0.02 ^ef^	0.41 ± 0.01 ^i^	0.30 ± 0.01 ^hi^	2.04 ± 0.10 ^jk^	0.44 ± 0.02 ^j^	0.56 ± 0.04 ^ij^	1.08 ± 0.02 ^no^
ZG989	0.27 ± 0.01 ^hi^	0.11 ± 0.01 ^ghi^	0.19 ± 0.001 ^gh^	0.67 ± 0.04 ^fg^	0.45 ± 0.02 ^c^	1.90 ± 0.01 ^k^	2.27 ± 0.16 ^a^	1.06 ± 0.05 ^bc^	1.74 ± 0.14 ^hi^
YG18	0.46 ± 0.02 ^c^	0.16 ± 0.02 ^de^	0.27 ± 0.01 ^d^	0.79 ± 0.02 ^de^	0.41 ± 0.01 ^d^	3.13 ± 0.02 ^de^	1.56 ± 0.09 ^cdef^	0.92 ± 0.02 ^ef^	1.72 ± 0.04 ^hi^
YG35	0.28 ± 0.01 ^h^	0.12 ± 0.01 ^fgh^	0.26 ± 0.01 ^de^	0.64 ± 0.03 ^g^	0.31 ± 0.01 ^gh^	2.66 ± 0.02 ^fgh^	0.41 ± 0.05 ^j^	0.97 ± 0.03 ^de^	2.41 ± 0.05 ^d^
YG36	0.32 ± 0.02 ^g^	0.24 ± 0.02 ^a^	0.25 ± 0.01 ^de^	0.66 ± 0.04 ^fg^	0.43 ± 0.02 ^cd^	2.65 ± 0.15 ^fgh^	1.71 ± 0.15 ^cd^	1.03 ± 0.03 ^cd^	2.28 ± 0.04 ^de^
YG43	0.06 ± 0.01 ^m^	0.11 ± 0.01 ^ghi^	0.15 ± 0.01 ^ij^	0.43 ± 0.01 ^i^	0.32 ± 0.02 ^gh^	1.33 ± 0.13 ^l^	0.47 ± 0.03 ^j^	0.35 ± 0.04 ^l^	1.49 ± 0.06 ^jk^
YG47	0.28 ± 0.01 ^h^	0.18 ± 0.02 ^bcd^	0.41 ± 0.02 ^a^	0.53 ± 0.03 ^h^	0.32 ± 0.01 ^gh^	3.44 ± 0.11 ^c^	1.73 ± 0.14 ^c^	0.85 ± 0.04 ^fg^	1.73 ± 0.05 ^hi^
YG48	0.55 ± 0.01 ^b^	0.16 ± 0.01 ^de^	0.33 ± 0.04 ^bc^	0.93 ± 0.07 ^ab^	0.56 ± 0.03 ^b^	3.19 ± 0.08 ^cd^	2.04 ± 0.02 ^b^	1.08 ± 0.04 ^bc^	2.25 ± 0.09 ^de^
JG20	0.64 ± 0.01 ^a^	0.15 ± 0.01 ^ef^	0.31 ± 0.01 ^c^	0.92 ± 0.02 ^b^	0.33 ± 0.02 ^fgh^	2.38 ± 0.09 ^hi^	1.71 ± 0.08 ^cd^	1.22 ± 0.06 ^a^	1.62 ± 0.05 ^ij^
JG39	0.36 ± 0.01 ^f^	0.18 ± 0.02 ^bcd^	0.30 ± 0.02 ^c^	0.66 ± 0.05 ^fg^	0.22 ± 0.01 ^k^	3.39 ± 0.06 ^cd^	1.28 ± 0.16 ^g^	0.73 ± 0.03 ^h^	1.44 ± 0.06 ^kl^
JG42	0.48 ± 0.02 ^c^	0.24 ± 0.04 ^a^	0.40 ± 0.01 ^a^	0.83 ± 0.03 ^cd^	0.26 ± 0.02 ^j^	2.62 ± 0.15 ^fgh^	1.66 ± 0.05 ^cde^	0.59 ± 0.02 ^i^	5.56 ± 0.24 ^a^
JG48	0.08 ± 0.01 ^m^	0.17 ± 0.01 ^bcde^	0.13 ± 0.01 ^j^	0.92 ± 0.04 ^b^	0.33 ± 0.02 ^efgh^	4.42 ± 0.19 ^b^	1.63 ± 0.12 ^cde^	0.83 ± 0.03 ^g^	3.51 ± 0.09 ^b^
ZG58	0.13 ± 0.01 ^kl^	0.14 ± 0.01 ^ef^	0.17 ± 0.01 ^hi^	0.84 ± 0.05 ^cd^	0.36 ± 0.01 ^ef^	2.70 ± 0.15 ^fg^	1.26 ± 0.09 ^gh^	0.60 ± 0.05 ^i^	1.20 ± 0.06 ^mn^
ZZ36	0.02 ± 0.01 ^n^	0.09 ± 0.01 ^i^	0.23 ± 0.02 ^ef^	0.55 ± 0.03 ^h^	0.33 ± 0.01 ^efgh^	2.31 ± 0.06 ^ij^	0.53 ± 0.03 ^j^	0.43 ± 0.06 ^kl^	1.39 ± 0.07 ^kl^
BG928	0.39 ± 0.01 ^e^	0.167 ± 0.013 ^cde^	0.20 ± 0.02 ^gh^	0.86 ± 0.02 ^bcd^	0.45 ± 0.02 ^c^	2.86 ± 0.10 ^ef^	1.51 ± 0.05 ^ef^	1.13 ± 0.04 ^b^	1.27 ± 0.06 ^lm^

Values with no letters in common are significantly different (*p* < 0.05). DW, dry weight of sample. Results are expressed as mean ± SD.

**Table 3:** Contents of individual phenolic compounds (mg/kg DW) in bound phenolic extracts of 22 dehusked foxtail millet varieties.

Dehusked Foxtail Millets	*p*-Hydroxybenzaldehyde	Syringic Acid	*Trans*-Ferulic Acid	*Cis*-Ferulic Acid	DFAs	*Trans*-*p*-Coumaric Acid	*Cis*-*p*-Coumaric Acid
JS1	0.43 ± 0.02 ^ghi^	0.45 ± 0.04 ^d^	104.68 ± 8.07 ^g^	35.18 ± 2.02 ^de^	14.95 ± 0.25 ^efg^	15.89 ± 2.51 ^e^	5.55 ± 0.82 ^b^
WG2	0.44 ± 0.03 ^ghi^	0.33 ± 0.02 ^e^	224.16 ± 14.78 ^cd^	21.01 ± 2.64 ^hi^	13.19 ± 2.08 ^hi^	82.57 ± 2.47 ^ghi^	2.52 ± 0.05 ^jk^
TS6	0.44 ± 0.03 ^ghi^	n.d.	209.53 ± 2.29 ^e^	47.64 ± 1.52 ^b^	14.63 ± 1.13 ^efg^	31.22 ± 3.95 ^l^	3.65 ± 0.24 ^fg^
CG9	0.48 ± 0.05 ^efg^	n.d.	357.54 ± 8.39 ^a^	28.89 ± 2.16 ^fg^	12.02 ± 0.54 ^j^	187.31 ± 5.56 ^a^	3.83 ± 0.13 ^f^
HG11	0.63 ± 0.05 ^c^	0.53 ± 0.04 ^c^	185.54 ± 6.07 ^f^	28.19 ± 1.29 ^g^	20.36 ± 0.76 ^ab^	85.09 ± 5.13 ^gh^	3.85 ± 0.16 ^f^
HG36	0.58 ± 0.01 ^cde^	0.43 ± 0.04 ^d^	226.30 ± 4.69 ^c^	22.90 ± 1.65 ^h^	14.45 ± 1.05 ^fg^	140.23 ± 3.41 ^c^	3.43 ± 0.28 ^g^
HG12	0.48 ± 0.04 ^efg^	0.47 ± 0.02 ^d^	181.55 ± 6.46 ^f^	24.06 ± 1.68 ^h^	14.01 ± 0.24 ^gh^	75.76 ± 3.87 ^i^	2.97 ± 0.07 ^h^
ZG15	0.37 ± 0.04 ^i^	n.d.	64.97 ± 2.69 ^k^	1.73 ± 0.24 ^k^	12.42 ± 0.24 ^ij^	11.76 ± 1.12 ^m^	2.21 ± 0.02 ^k^
ZG989	0.61 ± 0.04 ^c^	0.73 ± 0.02 ^b^	193.66 ± 3.06 ^f^	42.31 ± 1.54 ^c^	19.30 ± 0.51 ^bc^	106.84 ± 4.39 ^e^	6.78 ± 0.09 ^a^
YG18	0.53 ± 0.03 ^cdef^	0.92 ± 0.03 ^a^	222.73 ± 5.84 ^cde^	36.28 ± 3.43 ^d^	18.75 ± 0.90 ^c^	177.56 ± 5.67 ^b^	6.85 ± 0.11 ^a^
YG35	0.82 ± 0.03 ^a^	0.55 ± 0.01 ^c^	150.32 ± 6.82 ^g^	31.21 ± 2.55 ^efg^	13.01 ± 0.50 ^hij^	79.74 ± 1.63 ^hi^	4.21 ± 0.21 ^de^
YG36	0.72 ± 0.05 ^b^	0.55 ± 0.04 ^c^	192.36 ± 7.03 ^f^	32.28 ± 2.53 ^ef^	15.60 ± 0.29 ^e^	89.11 ± 1.67 ^fg^	3.90 ± 0.076 ^ef^
YG43	0.55 ± 0.04 ^cde^	0.45 ± 0.02 ^d^	209.73 ± 10.08 ^de^	23.05 ± 3.55 ^h^	14.00 ± 0.57 ^gh^	116.55 ± 5.06 ^d^	2.93 ± 0.05 ^h^
YG47	0.46 ± 0.03 ^fgh^	0.72 ± 0.03 ^b^	341.64 ± 8.91 ^a^	55.96 ± 2.72 ^a^	15.18 ± 0.38 ^ef^	171.38 ± 8.56 ^b^	5.07 ± 0.07 ^c^
YG48	0.39 ± 0.02 ^hi^	0.29 ± 0.01 ^e^	123.68 ± 7.36 ^h^	17.83 ± 1.61 ^i^	9.67 ± 0.39 ^k^	46.11 ± 1.79 ^j^	2.55 ± 0.09 ^ij^
JG20	0.43 ± 0.04 ^hi^	0.37 ± 0.02 ^e^	84.83 ± 12.06 ^jk^	6.95 ± 0.09 ^j^	6.99 ± 0.62 ^l^	40.64 ± 2.44 ^jk^	2.64 ± 0.16 ^hij^
JG39	0.53 ± 0.05 ^cdef^	n.d.	97.58 ± 5.29 ^j^	2.71 ± 0.16 ^k^	0.72 ± 0.08 ^n^	34.77 ± 1.35 ^kl^	n.d.
JG42	0.59 ± 0.04 ^defg^	n.d.	93.39 ± 2.07 ^j^	1.33 ± 0.41 ^k^	2.49 ± 0.22 ^m^	13.87 ± 1.01 ^m^	n.d.
JG48	0.57 ± 0.02 ^cd^	0.90 ± 0.04 ^a^	266.89 ± 11.17 ^b^	37.29 ± 2.28 ^d^	21.46 ± 0.81 ^a^	190.74 ± 2.39 ^a^	5.03 ± 0.63 ^c^
ZG58	0.55 ± 0.04 ^cde^	n.d.	145.33 ± 3.53 ^hi^	4.44 ± 0.52 ^jk^	10.36 ± 0.67 ^k^	95.71 ± 3.57 ^f^	2.85 ± 0.11 ^hi^
ZZ36	0.47 ± 0.02 ^hi^	n.d.	113.51 ± 6.56 ^hi^	18.76 ± 0.35 ^i^	15.65 ± 0.23 ^e^	94.59 ± 1.25 ^f^	3.44 ± 0.24 ^g^
BG928	0.54 ± 0.03 ^cde^	0.76 ± 0.04 ^b^	158.7 ± 2.04 ^g^	29.48 ± 0.63 ^fg^	17.04 ± 0.54 ^d^	142.61 ± 3.02 ^c^	4.25 ± 0.48 ^d^

Values with no letters in common are significantly different (*p* < 0.05). n.d., not detected. DW, dry weight of sample. Results are expressed as mean ± SD.

**Table 4:** Antioxidant activities by the DPPH, ABTS and FRAP assays of free and bound phenolic extracts of 22 dehusked foxtail millet varieties.

Dehusked Millets	DPPH (μmol TE/g DW)			ABTS (μmol TE/g DW)			FRAP (μmol TE/g DW)		
	Free	Bound	Total	Free	Bound	Total	Free	Bound	Total
JS1	0.58 ± 0.02 ^cde^	0.84 ± 0.04 ^i^	1.42 ± 0.06 ^i^	0.51 ± 0.02 ^l^	0.82 ± 0.06 ^l^	1.32 ± 0.08 ^l^	0.63 ± 0.02 ^k^	0.73 ± 0.014 ^k^	1.33 ± 0.02 ^i^
WG2	0.56 ± 0.08 ^cdef^	1.16 ± 0.05 ^cdef^	1.72 ± 0.13 ^cdef^	1.37 ± 0.11 ^cde^	1.62 ± 0.05 ^i^	2.99 ± 0.16 ^gh^	0.76 ± 0.02 ^efgh^	1.33 ± 0.04 ^ghij^	2.09 ± 0.06 ^efgh^
TS6	0.89 ± 0.04 ^a^	0.82 ± 0.03 ^defg^	1.71 ± 0.07 ^defg^	1.43 ± 0.09 ^cd^	1.94 ± 0.06 ^fg^	3.37 ± 0.15 ^cdef^	0.81 ± 0.02 ^de^	1.57 ± 0.03 ^cdefg^	2.38 ± 0.05 ^cde^
CG9	0.97 ± 0.02 ^cde^	1.56 ± 0.02 ^a^	2.53 ± 0.04 ^a^	1.22 ± 0.07 ^efgh^	2.18 ± 0.02 ^bc^	3.40 ± 0.09 ^cde^	0.63 ± 0.02 ^jk^	1.81 ± 0.11 ^b^	2.44 ± 0.13 ^bcd^
HG11	0.73 ± 0.05 ^b^	0.94 ± 0.03 ^efgh^	1.67 ± 0.08 ^efgh^	1.07 ± 0.02 ^hi^	2.14 ± 0.03 ^cd^	3.21 ± 0.05 ^defgh^	0.71 ± 0.01 ^hi^	1.39 ± 0.06 ^fghij^	2.18 ± 0.07 ^efgh^
HG36	0.62 ± 0.03 ^c^	1.21 ± 0.03 ^bcde^	1.83 ± 0.06 ^bcde^	1.13 ± 0.06 ^gh^	2.09 ± 0.04 ^de^	3.22 ± 0.10 ^defg^	1.19 ± 0.02 ^a^	1.66 ± 0.03 ^bcde^	2.85 ± 0.05 ^a^
HG12	0.53 ± 0.03 ^defg^	1.17 ± 0.04 ^efg^	1.70 ± 0.07 ^efg^	1.32 ± 0.06 ^cdef^	2.23 ± 0.01 ^b^	3.55 ± 0.07 ^c^	1.16 ± 0.02 ^a^	1.89 ± 0.17 ^ab^	2.86 ± 0.19 ^a^
ZG15	0.61 ± 0.03 ^cd^	1.32 ± 0.02 ^bc^	1.93 ± 0.05 ^bc^	1.22 ± 0.11 ^efgh^	1.86 ± 0.02 ^gh^	3.08 ± 0.13 ^gh^	0.72 ± 0.04 ^ghi^	1.49 ± 0.09 ^efghi^	2.21 ± 0.13 ^defgh^
ZG989	0.62 ± 0.04 ^cd^	0.86 ± 0.03 ^hi^	1.48 ± 0.07 ^hi^	1.27 ± 0.08 ^defg^	1.87 ± 0.03 ^fgh^	3.14 ± 0.11 ^fgh^	0.80 ± 0.03 ^def^	1.55 ± 0.023 ^defg^	2.35 ± 0.26 ^cdef^
YG18	0.60 ± 0.02 ^cd^	0.97 ± 0.04 ^fghi^	1.57 ± 0.06 ^fghi^	0.87 ± 0.09 ^j^	0.91 ± 0.06 ^k^	1.78 ± 0.15 ^k^	0.81 ± 0.02 ^de^	1.17 ± 0.02 ^j^	1.98 ± 0.04 ^gh^
YG35	0.53 ± 0.03 ^defg^	0.97 ± 0.04 ^ghi^	1.50 ± 0.07 ^ghi^	0.67 ± 0.04 ^k^	0.80 ± 0.05 ^l^	1.47 ± 0.09 ^l^	0.71 ± 0.03 ^ghi^	0.75 ± 0.03 ^k^	1.46 ± 0.06 ^i^
YG36	0.92 ± 0.03 ^a^	1.51 ± 0.02 ^a^	2.43 ± 0.05 ^a^	1.45 ± 0.08 ^c^	2.04 ± 0.04 ^e^	3.49 ± 0.12 ^c^	0.86 ± 0.02 ^d^	1.79 ± 0.03 ^bcd^	2.65 ± 0.05 ^abc^
YG43	0.50 ± 0.01 ^efg^	1.03 ± 0.04 ^fghi^	1.53 ± 0.05 ^fghi^	1.58 ± 0.03 ^b^	2.18 ± 0.03 ^bc^	3.76 ± 0.06 ^ab^	0.77 ± 0.02 ^efg^	1.63 ± 0.05 ^cdef^	2.43 ± 0.07 ^cde^
YG47	0.61 ± 0.04 ^cd^	1.05 ± 0.03 ^efjh^	1.66 ± 0.07 ^efgh^	1.79 ± 0.18 ^a^	2.09 ± 0.05 ^de^	3.88 ± 0.23 ^a^	0.97 ± 0.02 ^c^	1.81 ± 0.09 ^abc^	2.97 ± 0.11 ^a^
YG48	0.71 ± 0.05 ^b^	1.18 ± 0.14 ^bcd^	1.89 ± 0.19 ^bcd^	1.68 ± 0.12 ^ab^	2.11 ± 0.06 ^cde^	3.79 ± 0.18 ^ab^	0.85 ± 0.05 ^d^	2.06 ± 0.05 ^a^	2.91 ± 0.10 ^a^
JG20	0.61 ± 0.03 ^cd^	1.25 ± 0.22 ^bcde^	1.86 ± 0.25 ^bcde^	1.47 ± 0.11 ^c^	1.95 ± 0.05 ^f^	3.42 ± 0.16 ^cd^	0.93 ± 0.03 ^c^	1.51 ± 0.03 ^defgh^	2.44 ± 0.06 ^bcd^
JG39	0.60 ± 0.08 ^cd^	0.83 ± 0.03 ^i^	1.43 ± 0.11 ^i^	1.35 ± 0.05 ^cde^	1.82 ± 0.05 ^h^	3.17 ± 0.10 ^efgh^	0.72 ± 0.05 ^ghi^	1.57 ± 0.06 ^cdefg^	2.29 ± 0.11 ^defg^
JG42	0.46 ± 0.04 ^g^	1.06 ± 0.05 ^fghi^	1.52 ± 0.09 ^fghi^	1.18 ± 0.06 ^fgh^	1.88 ± 0.02 ^fgh^	3.06 ± 0.08 ^gh^	0.67 ± 0.01 ^ij^	1.54 ± 0.18 ^a^	2.16 ± 0.19 ^defgh^
JG48	0.48 ± 0.03 ^fg^	1.48 ± 0.05 ^b^	1.96 ± 0.08 ^b^	1.41 ± 0.01 ^cd^	2.37 ± 0.01 ^a^	3.78 ± 0.02 ^ab^	1.09 ± 0.02 ^b^	1.65 ± 0.11 ^bcde^	2.74 ± 0.13 ^ab^
ZG58	0.79 ± 0.07 ^b^	1.16 ± 0.02 ^b^	1.95 ± 0.09 ^b^	1.09 ± 0.01 ^h^	1.89 ± 0.02 ^fgh^	2.98 ± 0.03 ^h^	0.82 ± 0.01 ^de^	1.28 ± 0.06 ^ij^	2.15 ± 0.07 ^efgh^
ZZ36	0.33 ± 0.04 ^h^	1.03 ± 0.04 ^i^	1.36 ± 0.08 ^i^	0.91 ± 0.04 ^ij^	1.19 ± 0.03 ^j^	2.10 ± 0.07 ^j^	0.74 ± 0.07 ^fgh^	1.29 ± 0.46 ^hij^	2.03 ± 0.53 ^fgh^
BG928	0.73 ± 0.04 ^b^	0.80 ± 0.04 ^fghi^	1.53 ± 0.08 ^fghi^	0.89 ± 0.03 ^j^	1.58 ± 0.02 ^i^	2.47 ± 0.05 ^i^	0.71 ± 0.03 ^hi^	1.22 ± 0.03 ^j^	1.93 ± 0.06 ^h^

Results are expressed as mean ± SD. Values with no letters in common are significantly different (*p* < 0.05).

## Data Availability

The original contributions presented in this study are included in the article. Further inquiries can be directed to the corresponding author.

## Supplementary Materials

The following supporting information can be downloaded at: https://www.mdpi.com/article/10.3390/foods15091516/s1, Figure S1: UPLC chromatograms of the free and bound phenolics of the different foxtail millet varieties. Detection is shown at 280 nm for freephenolic compounds (A) and bound phenolic compounds (B); Table S1: List and color parameters of the 22 dehusked foxtail millet varieties; Table S2: Contents of proximate compounds of the 22 dehusked foxtail millet varieties.

## Abbreviations

The following abbreviations are used in this manuscript:

TPC	Total phenolic content
TFC	Total flavonoid content
TCC	Total carotenoid content
HCA	Hierarchical clustering analysis
FAE	Ferulic acid equivalents
RE	Rutin equivalents
TE	Trolox equivalents
DW	Dry weight of sample
