# Supplementary material for: Comparison of Nutritional and Functional Components and Antioxidant Activity of Different Foxtail Millet Varieties Grown in the Same Conditions
Source: Foods. 2026 Apr 27;15(9):1516. doi: 10.3390/foods15091516 (PMC13163399; doi:10.3390/foods15091516)
Supplement: Supplementary file 1 [file foods-15-01516-s001.zip › foods-4227477-supplementary.pdf]

Article

Comparison of Nutritional and Functional Componentss and Antioxidant Activity of Different Foxtail Millet Varieties Grown in the Same Conditions

Meng Li [1], Youyang Zhang [1], Junjie Hao [2], Runqiang Yang [3], Lei Luo [1] and Jinle Xiang [1,*]

[1] Faculty of Food & Bioengineering, Henan University of Science & Technology, Luoyang 471023, China; mengli6046@163.com (M.L.); youyang99215@163.com (Y.Z.); 13623896431@139.com (L.L.)

[2] Institute of Plant Protection, Henan Academy of Agricultural Sciences, Zhengzhou 450002, China; haojjds@163.com

[3] Faculty of Food Science & Technology, Nanjing Agricultural University, Nanjing 210000, China; yangrq@njau.edu.cn

* Correspondence: xjl5013@haust.edu.cn; Tel.: +86-152-3791-6981

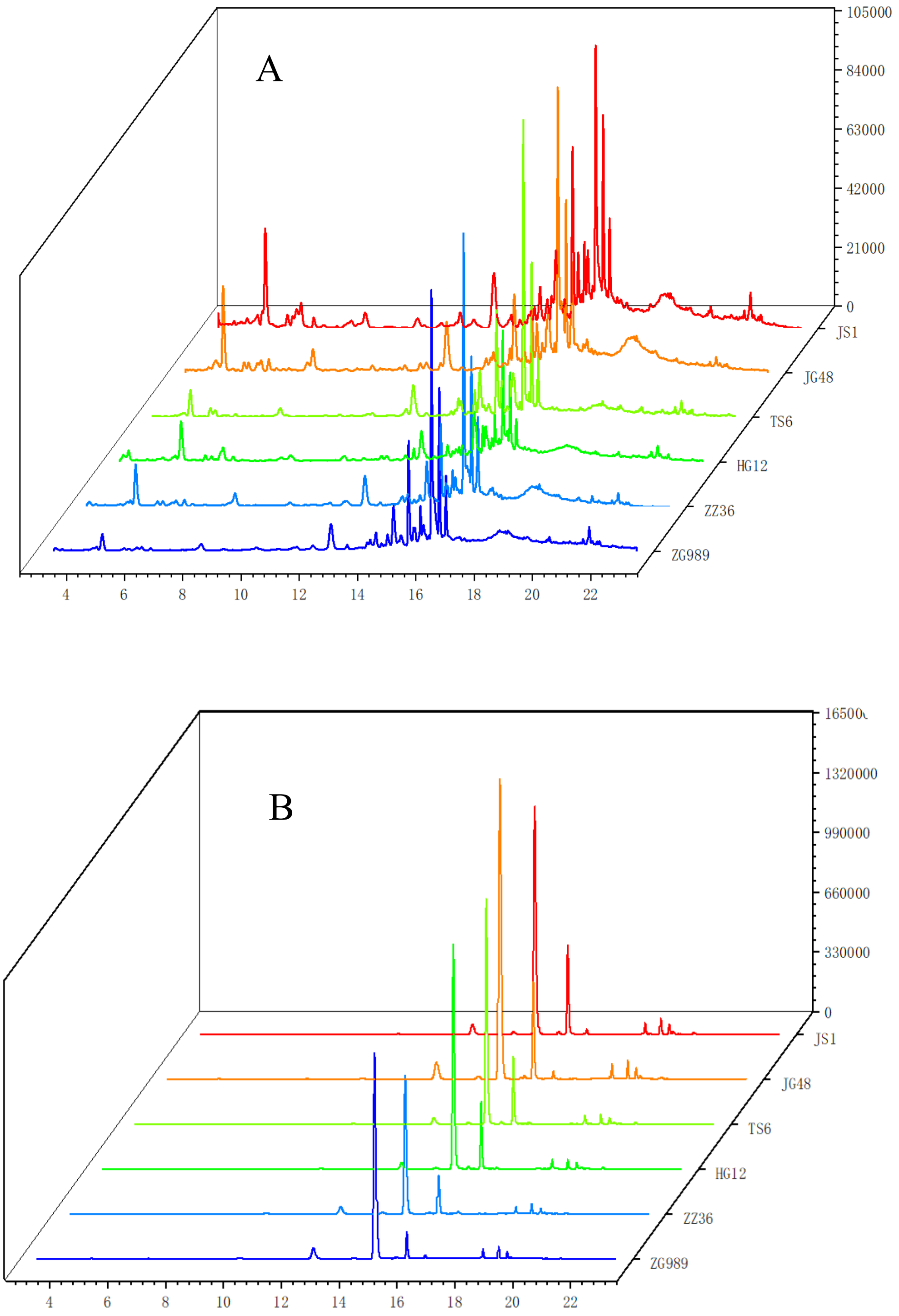

Figure S1. UPLC chromatograms of the free and bound phenolics of the different foxtail millet varieties. Detection was shown at 280 nm for freephenolic compounds (A) and bound phenolic compounds (B).

Table S1 List and color parameters of the 22 dehusked foxtail millet varieties.

**Dehusked foxtail millet varieties**	**Abbreviation**	**L***	**a***	**b***	**c***
Jinsu 1	JS1	73.72±0.98^{abcd}	4.66±0.16^{abcdefj}	34.53±2.19^{a}	13.87±0.67^{def}
Wangu 2	WG2	74.15±0.42^{abc}	3.99±0.32^{cdefgh}	31.53±1.09^{abcd}	17.32±0.31^{b}
Tiansu 6	TS6	74.05±0.36^{abc}	3.57±0.16^{h}	27.34±0.80^{gh}	18.23±0.32^{ab}
Canggu 9	CG9	71.47±0.50^{abcd}	5.48±0.39^{a}	32.31±0.85^{abc}	14.11±0.17^{cde}
Henggu 11	HG11	71.05±1.66^{bcd}	4.55±0.57^{abcdefgh}	30.19±2.40^{abcdefg}	15.79±0.81^{c}
Henggu 36	HG36	70.03±1.29^{d}	5.14±0.29^{ab}	30.71±2.39^{abcdefg}	17.23±0.98^{b}
Huagu 12	HG12	71.08±1.58^{bcd}	5.02±0.39^{abc}	29.37±0.64^{cdefg}	13.78±0.77^{ef}
Zhonggu 15	ZG15	72.86±1.46^{abcd}	3.79±0.22^{efgh}	29.22±2.40^{cdefg}	12.68±0.54^{ghi}
Zhonggu 989	ZG989	73.17±2.15^{abcd}	4.79±0.24^{abcde}	30.30±0.87^{abcdefg}	15.43±0.70^{c}
Yugu 18	YG18	73.32±2.21^{abcd}	3.99±0.58^{cdefgh}	30.89±2.44^{abcdef}	11.83±0.58^{ij}
Yugu 35	YG35	74.47±3.34^{abcd}	4.74±1.03^{abcdef}	31.77±0.71^{abcd}	12.34±0.46^{hij}
Yugu 36	YG36	72.64±2.96^{abcd}	4.35±0.68^{bcdefgh}	31.68±0.88^{abcd}	11.45±0.86^{j}
Yugu 43	YG43	72.47±0.35^{abcd}	4.21±0.37^{bcdefgh}	31.51±1.19^{abcd}	13.62±0.17^{fg}
Yugu 47	YG47	73.84±2.02^{abcd}	3.95±0.63^{defgh}	28.37±1.37^{defgh}	11.54±0.74^{j}
Yugu 48	YG48	74.50±1.92^{ab}	4.37±0.59^{bcdefgh}	31.41±0.68^{abcde}	17.84±0.78^{ab}
Jigu 20	JG20	72.08±0.75^{abcd}	4.56±0.32^{abcdefgh}	33.07±1.06^{ab}	13.69±0.99^{fg}
Jigu 39	JG39	72.73±1.69^{abcd}	4.71±0.77^{abcdef}	29.00±2.89^{cdefgh}	13.17±0.14^{fgh}
Jigu 42	JG42	70.77±4.54^{bcd}	3.73±0.13^{fgh}	27.95±1.83^{efgh}	17.58±0.46^{b}
Jigu 48	JG48	70.40±2.23^{cd}	4.97±1.05^{abcd}	30.41±1.60^{abcdefg}	14.92±0.47^{cd}
Zhaogu 58	ZG58	73.32±0.52^{abcd}	4.10±0.19^{cdefgh}	27.86±2.93^{fgh}	15.74±0.43^{c}
Zhongza 36	ZZ36	73.90±1.91^{abcd}	3.81±0.44^{gh}	25.57±1.37^{h}	18.77±0.73^{a}
Baogu 928	BG928	75.20±2.07^{a}	4.05±0.28^{cdefgh}	29.83±1.63^{bcdefg}	17.61±0.83^{b}

Results are expressed as mean ± SD. Values with no letters in common are significantly different ($p < 0.05$).

Table S2 Contents of proximate compounds of the 22 dehusked foxtail millet varieties

**Dehusked foxtail millets**	**Protein(g/100g)**	**Fat(g/100g)**	**Starch(g/100g)**	**Resistant Starch(g/100g)**	**Total carotenoid content(mg/kg)**
JS1	9.85 ± 0.13^{cdef}	2.46 ± 0.19^{hi}	66.00 ± 1.44^{def}	3.15 ± 0.23^{jk}	43.67 ± 4.19^{a}
WG2	9.82 ± 0.10^{cdef}	2.43 ± 0.14^{ij}	59.20 ± 3.08^{g}	3.61 ± 0.83^{ij}	33.71 ± 2.57^{de}
TS6	9.71 ± 0.28^{efg}	2.09 ± 0.12^{k}	72.76 ± 2.10^{b}	2.82 ± 0.21^{kl}	28.76 ± 1.10^{fgh}
CG9	8.65 ± 0.21^{jkl}	2.85 ± 0.16^{fg}	57.69 ± 1.80^{g}	5.65 ± 0.55^{bc}	32.56 ± 0.80^{def}
HG11	10.20 ± 0.06^{c}	2.30 ± 0.06^{ijk}	57.81 ± 0.72^{g}	5.86 ± 0.18^{b}	21.63 ± 0.92^{j}
HG36	10.17 ± 0.21^{cd}	3.07 ± 0.09^{ef}	63.89 ± 1.04^{f}	5.46 ± 0.31^{bc}	22.24 ± 0.65^{ij}
HG12	9.11 ± 0.14^{ih}	2.15 ± 0.10^{jk}	65.76 ± 1.62^{def}	3.95 ± 0.01^{ghi}	28.19 ± 2.23^{gh}
ZG15	10.13 ± 0.55^{cde}	3.14 ± 0.11^{de}	58.71 ± 0.47^{g}	3.56 ± 0.24^{ij}	26.08 ± 0.84^{hi}
ZG989	8.97 ± 0.21^{hijk}	2.48 ± 0.09^{hi}	58.88 ± 2.15^{g}	3.59 ± 0.08^{ij}	29.98 ± 1.23^{efgh}
YG18	8.92 ± 0.08^{hijkl}	2.83 ± 0.08^{fg}	79.64 ± 1.73^{a}	4.86 ± 0.05^{de}	40.64 ± 3.44^{ab}
YG35	8.85 ± 0.14^{ijkl}	2.45 ± 0.13^{hi}	65.36 ± 2.88^{def}	4.60 ± 0.22^{def}	31.67 ± 1.04^{efd}
YG36	8.50 ± 0.07^{lm}	1.76 ± 0.16^{l}	63.89 ± 1.04^{f}	4.49 ± 0.28^{defg}	36.05 ± 1.5^{cd}
YG43	8.78 ± 0.19^{ijkl}	2.14 ± 0.14^{k}	69.10 ± 2.12^{bcde}	5.08 ± 0.20^{cd}	22.55 ± 0.75^{ij}
YG47	8.17 ± 0.05^{m}	3.67 ± 0.17^{bc}	66.64 ± 1.13^{def}	2.34 ± 0.06^{lm}	38.99 ± 0.36^{bc}
YG48	8.54 ± 0.17^{klm}	2.30 ± 0.10^{ijk}	65.13 ± 2.98^{ef}	2.41 ± 0.13^{lm}	41.77 ± 1.71^{ab}
JG20	9.67 ± 0.22fg	2.20 ± 0.25^{ijk}	58.96 ± 1.93^{g}	2.37 ± 0.23^{lm}	33.61 ± 3.55^{de}
JG39	11.23 ± 0.24^{a}	3.41 ± 0.09^{cd}	69.18 ± 1.21^{bcd}	2.15 ± 0.04^{mn}	19.98 ± 1.44^{j}
JG42	9.33 ± 0.33^{gh}	3.80 ± 0.11^{b}	71.65 ± 3.21^{b}	4.31 ± 0.18^{efgh}	19.71 ± 1.89^{j}
JG48	10.72 ± 0.09^{b}	4.20 ± 0.19^{a}	71.21 ± 1.04^{bc}	4.74 ± 0.23^{de}	39.15 ± 1.98^{bc}
ZG58	9.74 ± 0.29^{def}	2.73 ± 0.13^{gh}	72.01 ± 1.86^{b}	3.86 ± 0.16^{hi}	30.45 ± 2.40^{efg}
ZZ36	9.08 ± 0.12^{hij}	2.90 ± 0.18^{efg}	72.80 ± 2.68^{b}	4.10 ± 0.27^{fghi}	20.21 ± 0.99^{j}
BG928	10.06 ± 0.30^{cdef}	3.08 ± 0.11^{ef}	67.59 ± 0.66^{cdef}	6.53 ± 0.27^{a}	31.03 ± 0.56^{efg}

Results are expressed as mean ± SD. Values with no letters in common are significantly different ($p < 0.05$).
